# Supplementary material for: Frequency, geographical distribution and outcomes of pit viper bites in Malaysia consulted to Remote Envenomation Consultancy Services (RECS) from 2017 to 2020
Source: PLoS Negl Trop Dis. 2023 Aug 16;17(8):e0011569. doi: 10.1371/journal.pntd.0011569 (PMC10461839; doi:10.1371/journal.pntd.0011569)
Supplement: S1 Table — (DOCX) [file pntd.0011569.s001.docx]

**SUPPORTING INFORMATION**

**Supplementary Material S1**

**S1 Table. The distribution of pit viper species according to States and Gazetteer for the year 2017-2020.**

| State | Gazetter | No of cases | Species |
| --- | --- | --- | --- |
| Johor | Kota Tinggi | 1 | *Trimeresurus purpureomaculatus* |
|  |  | 1 | *Tropidolaemus wagleri* |
|  | Pasir Gudang | 1 | *Trimeresurus purpureomaculatus* |
|  | Pengerang | 1 | *Trimeresurus purpureomaculatus* |
|  | Johor Bahru | 2 | *Trimeresurus purpureomaculatus* |
|  | Total | 6 |  |
| Selangor | Bukit Fraser | 1 | *Trimeresurus fucatus* |
|  | Sabak Bernam | 14 | *Trimeresurus purpureomaculatus* |
|  | Hutan Melintang | 1 | *Trimeresurus purpureomaculatus* |
|  |  | 1 | *Ovophis convictus* |
|  | Raub | 1 | *Trimeresurus fucatus* |
|  | Tanjung Karang | 10 | *Trimeresurus purpureomaculatus* |
|  | Cheras | 1 | *Trimeresurus flavomaculatus* |
|  | Kuala Selangor | 6 | *Trimeresurus purpureomaculatus* |
|  | Sungai Besar | 4 | *Trimeresurus purpureomaculatus* |
|  | Hutan Lipur Sg Tua | 1 | *Tropidolaemus wagleri* |
|  | Gombak | 2 | *Tropidolaemus wagleri* |
|  |  | 3 | *Trimeresurus purpureomaculatus* |
|  | Kuala Kubu Bharu | 1 | *Tropidolaemus wagleri* |
|  |  | 1 | *Trimeresurus hageni* |
|  |  | 3 | *Tropidolaemus wagleri* |
|  | Banting | 2 | *Trimeresurus purpureomaculatus* |
|  |  |  | *Trimeresurus purpureomaculatus* |
|  | Selayang | 1 | *Tropidolaemus wagleri* |
|  | Sungai Ayer Tawar | 1 | *Trimeresurus purpureomaculatus* |
|  | Teluk Intan | 1 | *Trimeresurus purpureomaculatus* |
|  | Hulu Selangor | 1 | *Trimeresurus hageni* |
|  | Kajang | 1 | *Trimeresurus purpureomaculatus* |
|  | Klang | 1 | *Trimeresurus purpureomaculatus* |
|  | Total | 58 |  |
| Terengganu | Kerteh | 1 | *Trimeresurus hageni* |
|  | Jerteh | 1 | *Tropidolaemus wagleri* |
|  | Kemaman | 3 | *Tropidolaemus wagleri* |
|  | Besut | 1 | *Trimeresurus wiroti* |
|  |  | 1 | *Tropidolaemus wagleri* |
|  | Paka | 1 | *Tropidolaemus wagleri* |
|  | Total | 8 |  |
| Sabah | Tuaran | 1 | *Tropidolaemus subannulatus* |
|  | Ranau | 1 | *Trimeresurus sabahi* |
|  |  | 1 | *Garthius chaseni* |
|  |  | 2 | *Tropidolaemus subannulatus* |
|  |  | 1 | *Trimeresurus sumatranus* |
|  | Kuala Penyu | 5 | *Tropidolaemus subannulatus* |
|  | Tambunan | 2 | *Trimeresurus borneensis* |
|  | Papar | 2 | *Trimeresurus borneensis* |
|  |  | 1 | *Tropidolaemus subannulatus* |
|  | Kota Marudu | 1 | *Tropidolaemus subannulatus* |
|  |  | 3 | *Trimeresurus sumatranus* |
|  | Membakut | 1 | *Trimeresurus borneensis* |
|  | Tamparuli | 1 | *Tropidolaemus subannulatus* |
|  | Kota Belud | 1 | *Trimeresurus sabahi* |
|  |  | 3 | *Tropidolaemus subannulatus* |
|  | Tawau | 1 | *Trimeresurus sumatranus* |
|  |  | 2 | *Tropidolaemus subannulatus* |
|  | Sandakan | 3 | *Tropidolaemus subannulatus* |
|  |  | 1 | *Trimeresurus borneensis* |
|  | Tungau | 1 | *Tropidolaemus subannulatus* |
|  | Sipitang | 2 | *Tropidolaemus subannulatus* |
|  | Kota Kinabalu | 1 | *Tropidolaemus subannulatus* |
|  | Bintangor | 1 | *Tropidolaemus subannulatus* |
|  | Kundasang | 1 | *Trimeresurus sabahi* |
|  |  | 1 | *Tropidolaemus subannulatus* |
|  | Kiulu | 1 | *Tropidolaemus subannulatus* |
|  | Menggatal | 1 | *Tropidolaemus subannulatus* |
|  | Total | 42 |  |
| Sarawak | Serian | 15 | *Tropidolaemus subannulatus* |
|  |  | 3 | *Trimeresurus borneensis* |
|  | Sri Aman | 7 | *Tropidolaemus subannulatus* |
|  | Similaju, Bintulu | 2 | *Trimeresurus sumatranus* |
|  |  | 8 | *Tropidolaemus subannulatus* |
|  | Bau | 3 | *Trimeresurus borneensis* |
|  |  | 6 | *Tropidolaemus subannulatus* |
|  | Singgai Bau | 1 | *Tropidolaemus subannulatus* |
|  | Ba Kelalan | 1 | *Trimeresurus sumatranus* |
|  | Dalat | 1 | *Tropidolaemus subannulatus* |
|  | Lawas | 4 | *Tropidolaemus subannulatus* |
|  |  | 3 | *Trimeresurus sumatranus* |
|  | Bakun | 1 | *Trimeresurus borneensis* |
|  | Hutan Lambir | 1 | *Tropidolaemus subannulatus* |
|  | Ulu Merapok | 1 | *Tropidolaemus subannulatus* |
|  | Simunjan | 1 | *Tropidolaemus subannulatus* |
|  | Long Banga | 1 | *Trimeresurus sumatranus* |
|  | Marudi | 2 | *Trimeresurus borneensis* |
|  |  | 3 | *Tropidolaemus subannulatus* |
|  | Kuching | 11 | *Tropidolaemus subannulatus* |
|  |  | 3 | *Trimeresurus borneensis* |
|  | Miri | 2 | *Trimeresurus borneensis* |
|  |  | 11 | *Tropidolaemus subannulatus* |
|  |  | 1 | *Trimeresurus sumatranus* |
|  | Pantu | 1 | *Tropidolaemus subannulatus* |
|  | Bintulu | 6 | *Tropidolaemus subannulatus* |
|  |  | 1 | *Trimeresurus borneensis* |
|  | Kanowit | 2 | *Tropidolaemus subannulatus* |
|  |  | 2 | *Trimeresurus borneensis* |
|  | Kapit | 1 | *Trimeresurus borneensis* |
|  |  | 2 | *Tropidolaemus subannulatus* |
|  | Batu Niah | 1 | *Tropidolaemus subannulatus* |
|  | Padawan | 1 | *Trimeresurus borneensis* |
|  | Tatau | 1 | *Tropidolaemus subannulatus* |
|  | Pakan | 1 | *Tropidolaemus subannulatus* |
|  | Sarikei | 3 | *Tropidolaemus subannulatus* |
|  | Limbang | 5 | *Tropidolaemus subannulatus* |
|  | Sibu | 2 | *Trimeresurus borneensis* |
|  | Betong | 1 | *Tropidolaemus subannulatus* |
|  | Lundu | 1 | *Tropidolaemus subannulatus* |
|  | Long San | 1 | *Trimeresurus borneensis* |
|  | Total | 124 |  |
| Kedah | Kulim | 1 | *Trimeresurus purpureomaculatus* |
|  | Baling | 4 | *Calloselasma rhodostoma* |
|  | Sik | 4 | *Calloselasma rhodostoma* |
|  | Yan | 1 | *Calloselasma rhodostoma* |
|  | Gerik | 1 | *Trimeresurus hageni* |
|  | Kuala Muda | 7 | *Trimeresurus purpureomaculatus* |
|  |  | 2 | *Calloselasma rhodostoma* |
|  | Sungai Petani | 1 | *Calloselasma rhodostoma* |
|  | Total | 21 |  |
| Kelantan | Tumpat | 1 | *Calloselasma rhodostoma* |
|  | Gua Musang | 1 | *Trimeresurus wiroti* |
|  |  | 1 | *Trimeresurus hageni* |
|  | Total | 3 |  |
| Melaka | Batang Melaka | 1 | *Tropidolaemus wagleri* |
|  | Klebang | 1 | *Trimeresurus purpureomaculatus* |
|  | Kuala Linggi | 1 | *Trimeresurus purpureomaculatus* |
|  | Total | 3 |  |
| Negeri Sembilan | Port Dickson | 2 | *Tropidolaemus wagleri* |
|  |  | 4 | *Trimeresurus purpureomaculatus* |
|  | Bahau | 1 | *Tropidolaemus wagleri* |
|  | Johol | 1 | *Tropidolaemus wagleri* |
|  | Sungai Linggi | 1 | *Trimeresurus purpureomaculatus* |
|  | Total | 9 |  |
| Pahang | Bentong | 1 | *Trimeresurus hageni* |
|  |  | 1 | *Trimeresurus wiroti* |
|  |  | 1 | *Trimeresurus fucatus* |
|  | Bertam | 1 | *Trimeresurus fucatus* |
|  | Betau | 1 | *Trimeresurus hageni* |
|  | Brinchang | 3 | *Ovophis convictus* |
|  | Bukit Tinggi | 1 | *Tropidolaemus wagleri* |
|  | Cameron Highlands | 10 | *Trimeresurus fucatus* |
|  |  | 1 | *Trimeresurus hageni* |
|  |  | 5 | *Ovophis convictus* |
|  |  | 10 | *Trimeresurus nebularis* |
|  | Dong | 1 | *Tropidolaemus wagleri* |
|  | Genting Highlands | 2 | *Trimeresurus fucatus* |
|  | Habu | 1 | *Trimeresurus wiroti* |
|  |  | 1 | *Trimeresurus fucatus* |
|  | Janda Baik | 1 | *Trimeresurus fucatus* |
|  |  | 1 | *Tropidolaemus wagleri* |
|  | Jerantut | 1 | *Tropidolaemus wagleri* |
|  |  | 1 | *Trimeresurus wiroti* |
|  | Kechau | 1 | *Tropidolaemus wagleri* |
|  | Kuala Lipis | 4 | *Tropidolaemus wagleri* |
|  | Kuantan | 2 | *Tropidolaemus wagleri* |
|  | Muadzam Shah | 2 | *Tropidolaemus wagleri* |
|  |  | 1 | *Trimeresurus hageni* |
|  | Raub | 3 | *Trimeresurus hageni* |
|  |  | 1 | *Tropidolaemus wagleri* |
|  | Ringlet | 1 | *Trimeresurus nebularis* |
|  |  | 1 | *Trimeresurus fucatus* |
|  |  | 5 | *Ovophis convictus* |
|  |  | 1 | *Trimeresurus hageni* |
|  | Sungai Lembing | 1 | *Trimeresurus sumatranus* |
|  | Tanah Rata | 1 | *Ovophis convictus* |
|  | Undocumented | 1 | *Trimeresurus fucatus* |
|  |  | 3 | *Trimeresurus hageni* |
|  |  | 1 | *Ovophis convictus* |
|  |  | 1 | *Trimeresurus nebularis* |
|  | Total | 74 |  |
| Penang | Kepala Batas | 2 | *Trimeresurus purpureomaculatus* |
|  | Bayan Lepas | 1 | *Trimeresurus purpureomaculatus* |
|  |  | 1 | *Tropidolaemus wagleri* |
|  | Penaga | 2 | *Trimeresurus purpureomaculatus* |
|  |  | 1 | *Calloselasma rhodostoma* |
|  | Nibong Tebal | 3 | *Trimeresurus purpureomaculatus* |
|  |  | 1 | *Calloselasma rhodostoma* |
|  |  | 2 | *Tropidolaemus wagleri* |
|  | Tasek Gelugor | 1 | *Trimeresurus purpureomaculatus* |
|  |  | 2 | *Calloselasma rhodostoma* |
|  | Seberang Perai | 9 | *Trimeresurus purpureomaculatus* |
|  |  | 9 | *Calloselasma rhodostoma* |
|  |  | 1 | *Tropidolaemus wagleri* |
|  | Prai | 1 | *Trimeresurus purpureomaculatus* |
|  | Permatang Pauh | 1 | *Trimeresurus purpureomaculatus* |
|  | Sungai Bakap | 3 | *Calloselasma rhodostoma* |
|  | Perai | 1 | *Trimeresurus purpureomaculatus* |
|  | Kulim | 1 | *Calloselasma rhodostoma* |
|  | Pinang Tunggal | 1 | *Calloselasma rhodostoma* |
|  | South Seberang Perai | 1 | *Trimeresurus purpureomaculatus* |
|  | Balik Pulau | 1 | *Calloselasma rhodostoma* |
|  | Kubang Semang | 1 | *Calloselasma rhodostoma* |
|  | Parit Buntar | 1 | *Trimeresurus purpureomaculatus* |
|  | Pulau Pinang | 1 | *Trimeresurus purpureomaculatus* |
|  | Butterworth | 1 | *Trimeresurus purpureomaculatus* |
|  | Simpang Ampat | 1 | *Trimeresurus purpureomaculatus* |
|  | Undocumented | 1 | *Trimeresurus fucatus* |
|  |  | 1 | *Trimeresurus purpureomaculatus* |
|  | Total | 59 |  |
| Perak | Sungai Sumun | 1 | *Trimeresurus purpureomaculatus* |
|  | Tapah | 4 | *Trimeresurus hageni* |
|  |  | 5 | *Tropidolaemus wagleri* |
|  |  | 1 | *Trimeresurus wiroti* |
|  |  | 1 | *Trimeresurus fucatus* |
|  | Kuala Sepetang | 2 | *Trimeresurus purpureomaculatus* |
|  | Batang Padang | 1 | *Tropidolaemus wagleri* |
|  |  | 1 | *Tropidolaemus wagleri* |
|  | Jalan Pahang | 1 | *Trimeresurus fucatus* |
|  | Nibong Tebal | 7 | *Trimeresurus purpureomaculatus* |
|  | Bukit Larut | 1 | *Tropidolaemus wagleri* |
|  | Manjung | 3 | *Trimeresurus purpureomaculatus* |
|  | Teluk Emas | 1 | *Calloselasma rhodostoma* |
|  | Kuari Sungai Jelutong | 1 | *Tropidolaemus wagleri* |
|  | Kelian Gunung | 1 | *Tropidolaemus wagleri* |
|  | Kerian | 4 | *Trimeresurus purpureomaculatus* |
|  |  | 1 | *Tropidolaemus wagleri* |
|  | Bagan Serai | 1 | *Trimeresurus purpureomaculatus* |
|  | Perak | 1 | *Trimeresurus purpureomaculatus* |
|  | Selama | 7 | *Tropidolaemus wagleri* |
|  |  | 13 | *Calloselasma rhodostoma* |
|  | Kampar | 3 | *Tropidolaemus wagleri* |
|  | Kinta | 1 | *Tropidolaemus wagleri* |
|  | Gerik | 5 | *Tropidolaemus wagleri* |
|  | Tambun | 1 | *Tropidolaemus wagleri* |
|  | Banding | 1 | *Trimeresurus sumatranus* |
|  | Mahang | 3 | *Calloselasma rhodostoma* |
|  | Rantau Panjang | 1 | *Tropidolaemus wagleri* |
|  |  | 1 | *Calloselasma rhodostoma* |
|  |  | 1 | *Tropidolaemus wagleri* |
|  | Ijok | 1 | *Tropidolaemus wagleri* |
|  | Taiping | 1 | *Trimeresurus hageni* |
|  |  | 1 | *Tropidolaemus wagleri* |
|  | Kuala Kurau, Kerian | 1 | *Trimeresurus purpureomaculatus* |
|  | Chemor | 1 | *Tropidolaemus wagleri* |
|  | Bagan Datoh | 3 | *Trimeresurus purpureomaculatus* |
|  | Legap | 1 | *Tropidolaemus wagleri* |
|  | Parit Buntar | 2 | *Trimeresurus purpureomaculatus* |
|  | Teluk Intan | 4 | *Trimeresurus purpureomaculatus* |
|  | Hilir Perak | 1 | *Trimeresurus purpureomaculatus* |
|  | Sitiawan | 1 | *Calloselasma rhodostoma* |
|  |  | 1 | *Trimeresurus purpureomaculatus* |
|  | Simpang Pulai | 1 | *Trimeresurus hageni* |
|  | Malim Nawar | 1 | *Tropidolaemus wagleri* |
|  | Gopeng | 2 | *Tropidolaemus wagleri* |
|  | Hulu Perak | 1 | *Tropidolaemus wagleri* |
|  | Gurun | 1 | *Trimeresurus hageni* |
|  | Sungkai | 1 | *Trimeresurus fucatus* |
|  | Larut | 1 | *Tropidolaemus wagleri* |
|  | Serdang | 1 | *Calloselasma rhodostoma* |
|  | Pahang | 1 | *Trimeresurus fucatus* |
|  | Kuala Kangsar | 2 | *Tropidolaemus wagleri* |
|  | Batu Gajah | 1 | *Tropidolaemus wagleri* |
|  | Batu Kurau | 1 | *Trimeresurus hageni* |
|  | Hutan Melintang | 2 | *Trimeresurus purpureomaculatus* |
|  | Sungai Siput | 1 | *Tropidolaemus wagleri* |
|  | Padang Rengas | 1 | *Tropidolaemus wagleri* |
|  | Undocumented | 1 | *Trimeresurus purpureomaculatus* |
|  |  | 1 | *Tropidolaemus wagleri* |
|  |  | 1 | *Trimeresurus fucatus* |
|  |  | 3 | *Calloselasma rhodostoma* |
|  |  | 1 | *Ovophis convictus* |
|  | Total | 120 |  |
| Perlis | Undocumented | 1 | *Trimeresurus venustus* |
| Total |  | 523 |  |
