# Supplementary material for: Frequency, geographical distribution and outcomes of pit viper bites in Malaysia consulted to Remote Envenomation Consultancy Services (RECS) from 2017 to 2020
Source: PLoS Negl Trop Dis. 2023 Aug 16;17(8):e0011569. doi: 10.1371/journal.pntd.0011569 (PMC10461839; doi:10.1371/journal.pntd.0011569)
Supplement: Attachment — Submitted filename: Comments.pdf [file pntd.0011569.s002.pdf]

Frequency, Geographical Distribution and Outcomes of Pit Viper Bite Envenomation in Malaysia

This study provides important conclusions to the health care system in order to plan proper management of snakebites, particularly having sufficient stocks of antivenom.

But, there are several issues in the manuscript.

Method is not clear. Authors have not mentioned sufficient information in this section. Therefore, it should be rewritten including the following concerns,

-although it was mentioned in Introduction about a collaboration of MyBIS with RECS, it was not mentioned in Method how data were collected from the system for this retrospective study. Authors should mention clearly the details regarding this information system.

-Mention about the administrative approval

-Usually, in hospital clinic records, species names have not been recorded and common names are mentioned instead e.g. Green pit viper, Russell's viper etc. unless if a well-planned prospective study is done. Therefore, in this situation, how did authors identify species of pit vipers

Heading

It is better to remove the word envenomation and the suggested heading is,

Frequency, Geographical Distribution and Outcomes of Pit Viper Bites in Malaysia

Introduction

Line 54-56

write short sentences

Line 56-57

meaning is not clear

Line 68-72

How many pit viper species in Malaysia? Are there endemic species?

Methods

Line 88

Mention ERC reference No.

Line 89

Considering the retrospective nature of this study, explain universal sampling method of the study

Line 92-93

As this is a retrospective study, explain “The species confirmation was verified by RECS experts during each consultation based on the specimen brought to the hospital”

Are all these data available in the patients’ clinical records?

Line 93

How species identification was possible by using a picture?

Results

Table 2

How the species level identification was done in this study?

Line 132-133

What are OSMI and NKAV?

Discussion

184-185

….no antivenom is required for bites from *Tropidolaemus* species in the peninsula and east Malaysia.

Line 192

US??

Line 296

…diagnoses….

References

Please replace very old references e.g 4, 7, 8 with recent ones

Tables

Table 1

It is not clear following contents

Not documented 1
median 38
Minimum 0
Max 85

Please use a clear table format

Figures

Letters are not clear (blurred) in all figures
