# Supplementary material for: Frequency, geographical distribution and outcomes of pit viper bites in Malaysia consulted to Remote Envenomation Consultancy Services (RECS) from 2017 to 2020
Source: PLoS Negl Trop Dis. 2023 Aug 16;17(8):e0011569. doi: 10.1371/journal.pntd.0011569 (PMC10461839; doi:10.1371/journal.pntd.0011569)
Supplement: Attachment — Submitted filename: PNTD-D-23-006014_Rev_230606.docx [file pntd.0011569.s003.docx]

# Review Comments to the Editor

I reviewed this manuscript thoroughly. This manuscript can be considered with a minor revision. This article addresses the knowledge gap on identified pitviper bites in Malaysia. This paper can be reference for antivenom design and distribution in addition to the formulation of prevention strategies. It is a plus for PLOS Negl Trop Dis.

This paper can be outstanding in its discipline for Malaysia where policy makers can use its findings to improve management of snakebites. The methodology is sufficient to allow reproducing the study. This manuscript is well organized and written clearly. I have mentioned my feedbacks to authors explicitly in a separate section so that authors will be able to improve this manuscript and publish it in PLOS Neglected Tropical Diseases.

# Review Comments to the Author

# General

Although authors have written introduction, methods, results and discussion sections well, they have not studied literature enough. Reid and Theakston (1983) mentioned that a prospective hospital-based study of snakebite cases in Malaysia confirmed that snake bite is occupational hazard mainly among males during daylight particularly in a rural Malaysia. But, findings of majority of non-occupational hazards are contrary to this report. Authors did not discuss well in the discussion section in support of published literature.

# Specific

## Introduction

This section is well organized with convincing arguments. However, I have suggested you minor improvement below.

Line 50-52: It is important to keep in mind that a high incidence of snake bite is often related to the prevalence of particular snake species. For example, *E. carinatus* are abundant in West Africa, Pakistan, and north-west India, *A. rhodostoma* in south-east Asia, and *V. russelli* in Burma and other area of Asia where they have high incidence of snakebite. So, studying epidemiology of snakebite in regions having abundance of particular venomous snakes cannot be regionally or nationally representative. Authors might have written above paragraph in this context. But, this looks that reference number one has remarked about the limitation of the study design. So, I suggest revising these lines as:

Please, replace "The actual burden from snakebite envenomation in affected countries is poorly understood mainly due to lack of properly designed population surveys looking at incidence, morbidity, and mortality [1]."

with

"The actual burden from snakebite envenomation in affected countries is poorly understood mainly due to lack of properly designed population surveys. For example, a survey carried in a couple of snakebite prone villages of eastern lowlands of Nepal reported unexpectedly higher incidence and mortality of snakebite cases [1] than those reported from comparable snakebite prone areas in Nepal (Pandey 2007), Bangladesh (....reference), and India (....reference)."

[1. Sharma SK, Chappuis F, Jha N, Bovier PA, Loutan L, Koirala S. Impact of snake bites

and determinants of fatal outcomes in southeastern Nepal. Am J Trop Med Hyg. 2004;71:234–238.

**Pandey DP. 2007.** Epidemiology of snakebites based on field survey in Chitwan and Nawalparasi Districts, Nepal. Journal of Medical Toxicology 3: 164–168.

Line 52-57: "...Literature searches on snakebite epidemiology in Malaysia are ... Sabah and Sarawak. **"**

**Reviewer:** Study of snakebite epidemiology in Malaysia began as early as in 1960s (Reid HA, Thean P, Martin W. 1963. Epidemiology of snake bite in north Malaya. British medical journal 1: 992.). There are several other epidemiological study in Malaysia. So, please, replace these lines ..."Literature searches on snakebite epidemiology in Malaysia are scarce and most diagnoses were unverified, especially regarding the identification of the snake species [2–4]."

with

..."Although studies on snakebite epidemiology were carried in Malaysia since 1960s, most diagnoses of snake species and their bites were unverified [2–4, please, add other relevant references herein]."

Line 67-72: Please, replace "Documented pit viper species include *Trimeresurus purpureomaculatus, Trimeresurus hageni, Trimeresurus*

*nebularis, Trimeresurus malcomi, Trimeresurus sabahi fucatus , Trimeresurus borneensis,*

*Trimeresurus buniana, Trimeresurus Sabahi sabahi, Trimeresurus sumatranus, Trimeresurus*

*venustus, Trimeresurus wiroti, Tropidolaemus subannulatus, Tropidolaemus wagleri,*

*Calloselasma rhodostoma, Garthius chaseni, Ovophis convictus.*"

with

" Documented pit viper species include *Trimeresurus purpureomaculatus, T. hageni, T.* *nebularis, T. malcomi, T. sabahi fucatus, T. borneensis, T. buniana, T. sabahi sabahi, T. sumatranus, T. venustus, T. wiroti, Tropidolaemus subannulatus, Tropidolaemus wagleri,*

*Calloselasma rhodostoma, Garthius chaseni, Ovophis convictus.*".

Line 69:

please,... remove extra space in " *Trimeresurus sabahi fucatus* and comma".

Line 70:

please replace " *Trimeresurus Sabahi sabahi* " with " *Trimeresurus sabahi sabahi* "

## Methods

This section is well presented. However, I have little concern about snake identification. Ashar et al. (2016) established a Remote Envenomation Consultation Service, which authors used during this cohort study. Are these identification based on morphometric analysis or both morphometric as well as DNA barcode analyses? Reliable methods used while identifying the snakes involved in bite to human subject could make this section more convincing. It is because several pitviper species have species complexes. Aren't there species complex in pitviper of Malaysia?

[Ashar AM, Lam MC, Zainudin S, Ismail AK. 2016. A preliminary study on the decision support mobile application for remote snakebite management consultation in Malaysia. Pages 020086-020081–020086. AIP Conference Proceedings: American Institute of Physics Publishing.]

## Results

**Table 1. Demographic**

please, use frequency instead of symbol f or n

percent instead of % .... Or, make subheadings of this table like in Table 2.

## Discussion

Line 159–162: "... bite cases in this study involved local citizens and occurred during non-occupational activity possibly because pit vipers are mostly nocturnal and outdoor are mainly performed during daytime. It is uncommon to find pit vipers entering houses, and in this study only 2.9 % of cases occurred indoors."

Rev: Reid and Theakston (1983) mentioned that a prospective hospital-based study of snakebite cases in Malaysia confirmed that snake bite is occupational hazard mainly among males during daylight particularly in a rural Malaysia. But, your findings are contrary to this report which you did not discuss well in the discussion section in support of previous reports and recent findings which are in favour of your report.

Line 142-143: "... the actual burden because many victims never reach primary care facilities and are therefore unreported."

Rev: If so, where do they receive treatment services? Do they visit to traditional healers like in other Asian and African countries? Please, mention it following "....unreported.".

Line 144: "... The Malaysian literature on snakebite epidemiology is scarce [14].".

Rev: please replace "... The Malaysian literature on snakebite epidemiology is scarce [14]."

with

"... The Malaysian literature on proven snakebite epidemiology is scarce [14]."

Line 200: Please, insert full stop and They between "*Bitis arietans*" and "have been referred to" .

## Referencing

Line 282

"18. Ismail AK, Adnan A, Hassan A, Mohamed Z, Daud R. RECS-01-01. 2014.". Please, make this reference complete with title having full form of the abbreviation RECS-

Is this original title or did you abbreviate it?

Reid, H. A., & Theakston, R. D. G. (1983). The management of snake bite. *Bulletin of the World Health Organization, 61*(6), 885–895.
