# Supplementary material for: Frequency, geographical distribution and outcomes of pit viper bites in Malaysia consulted to Remote Envenomation Consultancy Services (RECS) from 2017 to 2020
Source: PLoS Negl Trop Dis. 2023 Aug 16;17(8):e0011569. doi: 10.1371/journal.pntd.0011569 (PMC10461839; doi:10.1371/journal.pntd.0011569)
Supplement: Attachment — Submitted filename: Response to Reviewer.docx [file pntd.0011569.s004.docx]

Responses to Reviewers

Frequency, Geographical Distribution and Outcomes of Pit Viper Bite Envenomation in Malaysia

**Reviewer 1**

This study provides important conclusions to the health care system in order to plan proper management of snakebites, particularly having sufficient stocks of antivenom. But, there are several issues in the manuscript. Method is not clear. Authors have not mentioned sufficient information in this section. Therefore, it should be rewritten including the following concerns, although it was mentioned in Introduction about a collaboration of MyBIS with RECS, it was not mentioned in Method how data were collected from the system for this retrospective study. Authors should mention clearly the details regarding this information system. Mention about the administrative approval

***Response:*** *Thank you for the comment, we have included in the method on how data was collected from RECS consultation log.*

Usually, in hospital clinic records, species names have not been recorded and common names are mentioned instead e.g. Green pit viper, Russell’s viper etc. Unless if a well-planned prospective study is done. Therefore, in this situation, how did authors identify species of pit vipers

***Response:*** *We acknowledged the issue with hospital/clinic records, that’s the reason why RECS consultation plays an important role for snake related injuries in Malaysia. The data in this study is not taken from hospital/clinic records. As mentioned in the introduction and methodology, the data is collected from RECS consultation log. The species confirmation was verified by RECS experts during each consultation based on the specimen brought to the hospital or the picture of the actual specimen taken.*

# **Heading**

It is better to remove the word envenomation and the suggested heading is, Frequency, Geographical Distribution and Outcomes of Pit Viper Bites in Malaysia

***Response:*** *Thank you, we have changed the title accordingly.*

**Introduction**

Line 54-56 write short sentences
Line 56-57 meaning is not clear

***Response:*** *Thank you, the long sentence has been truncated and the meaning for the states of Sabah and Sarawak has been clarified.*

Line 68-72 How many pit viper species in Malaysia? Are there endemic species?

***Response:*** *Thank you, currently there are 16 species of pit viper in Malaysia. However, not all species are endemic in all the states of Peninsular or East Malaysia.*

# **Methods**

Line 88 Mention ERC reference No.
Line 89 Considering the retrospective nature of this study, explain universal sampling method of the study
Line 92-93 As this is a retrospective study, explain “The species confirmation was verified by RECS experts during each consultation based on the specimen brought to the hospital” Are all these data available in the patients’ clinical records?
Line 93 How species identification was possible by using a picture?

***Response:*** *Thank you, the ERC research reference number has been included. The explanation for how RECS consultation and how species identification was performed are explained in the last paragraph of the introduction and in the methodology.*

# **Results**

Table 2 How the species level identification was done in this study?

***Response:*** *The species identification was verified by RECS consultants and documented in each case diagnosis in the RECS consultation log following the consultation.*

*This is the list of diagnosis used in Malaysia following RECS consultation and in the Ministry of Health Snakebite Management Guidelines (2017) and proposed for ICD10 classification for snake related injuries in Malaysia.*
*A working diagnosis for Snake Related Injuries (SRI) diagnosis when a victim arrives in a healthcare facility:*
*1. Identified snakebite (provide the name of the snake species)*
*2. Unidentified snakebite*
*3. Venom ophthalmia (always related to Naja sumatrana in Malaysia)*
*For no 1, the specimen or the picture of the actual snake that cause the injury mist be available for verification (snakeID kit is not available in Malaysia yet).*
*For no 2, the patient or other eyewitness must onserved that a snake caused the bite injury, but the species is not available for verification (snakeID kit results may have a role in the future)*
*For both working dx, envenomation may occur for bites from venomous species as evident by close serial observation of clinical signs, symptoms and relevant lab investigation results.*

*Note: A final diagnosis could either be with or without significant envenomation.*

*Other working diagnosis for consideration are:*

*1. Identified animal bite/sting other than SRI (provide type/species of animal)|*
*2. Unidentified animal bite/sting*
*3. Unidentified cause of injury.*
*For no 2, the patient or other eyewitness must observed that an animal caused the bite injury, but the type of animal is not observed and not available for verification.*
*For both no 2 & 3, the final diagnosis may become evident following a close serial observation of clinical signs, symptoms and relevant lab investigation results.*
*For 1, 2 & 3, the final diagnosis could either be with or without significant envenomation.*
*'Suspected snakebite' is NOT a verified clinical diagnosis.*

Line 132-133 What are OSMI and NKAV?

***Response:*** *Thank you, QSMI stands for Queen Saovabha Memorial Institute and NKAV stands for Naja Kaouthia antivenom. Changes have been made.*

# **Discussion**

184-185 ….no antivenom is required for bites from *Tropidolaemus* species in the peninsula and east Malaysia.

***Response:*** *Thank you, the changes have been made.*

Line 192 US??
Line 296 …diagnoses….

*Response: Thank you, changes have been made.*

**References**

Please replace very old references e.g 4, 7, 8 with recent ones

*Response: These references were in referenced to the limited snake bite epidemiology study in Malaysia in the past. The publication of this study would be the most reliable and current.*

**Tables**

Table 1: It is not clear following contents, Not documented 1 median 38 Minimum 0, Max 85
Please use a clear table format

***Response:*** *Thank you. Changes have been made.*

# **Figures**

Letters are not clear (blurred) in all figures

*Response: Thank you. All figures have been prepared and uploaded according to the specifications provided by the journal.*

**Reviewer 2**

# **Review Comments to the Author**

Although authors have written introduction, methods, results and discussion sections well, they have not studied literature enough. [Reid and Theakston (1983)](bookmark://_ENREF_1) mentioned that a prospective hospital-based study of snakebite cases in Malaysia confirmed that snake bite is occupational hazard mainly among males during daylight particularly in a rural Malaysia. But, findings of majority of non-occupational hazards are contrary to this report. Authors did not discuss well in the discussion section in support of published literature.

***Response:*** *Thank you, the reference quoted by Reid and Theakston (1983) on page 886 first paragraph under subtopic epidemiology, mentioned “A prospective 3-year study based on hospital cases in Malaysia confirmed that snake bite is mainly a rural and occupational hazard, most bites occurring in males during daylight, when more people are exposed to risk. The severity of poisoning showed no significant variation with the time of the bite (during the day or after dark), the part of the body bitten, the age of the victim, or the breeding habits of the snakes.” However, no specific reference was provided for such study in this article. Therefore, we could not quote it for our study. Most likely the study was conducted pre-1980s when Malaysia was transitioning from mainly an agriculturally based economy to modern industries.*

*“There appears to be a variety of situations of snakebite in Malaysia, not just related to occupation. The situations and frequency of incidents may ﬂuctuate with the diversity of human activities and the changing of lifestyles. These associations are dynamic, yet with the modernization of the agricultural sector, the chance for unfavorable contact is expected to reduce. Unfortunately, as the human population increases, so do the size of residential and industrial areas. These expansions encroach into and directly decrease the continued existence and quality of natural habitats."*

*Ref: Ismail AK. Snakebite and Envenomation Management in Malaysia. In: Gopalakrishnakone P, Faiz A, Fernando R, Gnanathasan CA, Habib AG, Yang C-C, editors. Clinical Toxinology in Asia Pacific and Africa. Dordrecht: Springer Netherlands; 2015. pp. 71–102. doi:10.1007/978-94-007-6386-9_54*

## **Introduction**

This section is well organized with convincing arguments. However, I have suggested you minor improvement below.

Line 50-52: It is important to keep in mind that a high incidence of snake bite is often related to the prevalence of particular snake species. For example, *E. carinatus* are abundant in West Africa, Pakistan, and north-west India, *A. rhodostoma* in south-east Asia, and *V. russelli* in Burma and other area of Asia where they have high incidence of snakebite. So, studying epidemiology of snakebite in regions having abundance of particular venomous snakes cannot be regionally or nationally representative. Authors might have written above paragraph in this context. But, this looks that reference number one has remarked about the limitation of the study design. So, I suggest revising these lines as:

Please, replace "The actual burden from snakebite envenomation in affected countries is poorly understood mainly due to lack of properly designed population surveys looking at incidence, morbidity, and mortality [1]." with "The actual burden from snakebite envenomation in affected countries is poorly understood mainly due to lack of properly designed population surveys. For example, a survey carried in a couple of snakebite prone villages of eastern lowlands of Nepal reported unexpectedly higher incidence and mortality of snakebite cases [1] than those reported from comparable snakebite prone areas in Nepal (Pandey 2007), Bangladesh (....reference), and India (....reference)."

[1] Sharma SK, Chappuis F, Jha N, Bovier PA, Loutan L, Koirala S. Impact of snake bites

and determinants of fatal outcomes in southeastern Nepal. Am J Trop Med Hyg. 2004;71:234–238.

**Pandey DP. 2007.** Epidemiology of snakebites based on field survey in Chitwan and Nawalparasi Districts, Nepal. Journal of Medical Toxicology 3: 164–168.

***Response:*** *Thank you for the suggestion. We have added more relevant reference for the region. This sentence is clear and well understood as referred by WHO. The references provided are relevant to this study.*

Line 52-57: "...Literature searches on snakebite epidemiology in Malaysia are ... Sabah and Sarawak. **"**

Reviewer: Study of snakebite epidemiology in Malaysia began as early as in 1960s (Reid HA, Thean P, Martin W. 1963. Epidemiology of snake bite in north Malaya. British medical journal 1: 992.). There are several other epidemiological study in Malaysia. So, please, replace these lines ..."Literature searches on snakebite epidemiology in Malaysia are scarce and most diagnoses were unverified, especially regarding the identification of the snake species [2–4]." with ..."Although studies on snakebite epidemiology were carried in Malaysia since 1960s, most diagnoses of snake species and their bites were unverified [2–4, please, add other relevant references herein]."

***Response:*** *Thank you. The other relevant references [5-8] are already provided in the next sentence.*

Line 67-72: Please, replace "Documented pit viper species include *Trimeresurus purpureomaculatus, Trimeresurus hageni, Trimeresurus* *nebularis, Trimeresurus malcomi, Trimeresurus sabahi fucatus , Trimeresurus borneensis,* T*rimeresurus buniana, Trimeresurus Sabahi sabahi, Trimeresurus sumatranus, Trimeresurus* *venustus, Trimeresurus wiroti, Tropidolaemus subannulatus, Tropidolaemus wagleri,* C*alloselasma rhodostoma, Garthius chaseni, Ovophis convictus.*" With "Documented pit viper species include *Trimeresurus purpureomaculatus, T. hageni, T.* *nebularis, T. malcomi, T. sabahi fucatus, T. borneensis, T. buniana, T. sabahi sabahi, T. sumatranus, T. venustus, T. wiroti, Tropidolaemus subannulatus, Tropidolaemus wagleri,* C*alloselasma rhodostoma, Garthius chaseni, Ovophis convictus.*".

Line 69: please,... remove extra space in " *Trimeresurus sabahi fucatus* and comma".

Line 70: please replace " *Trimeresurus Sabahi sabahi* " with " *Trimeresurus sabahi sabahi* "

***Response:*** *Thank you. Changes have been made accordingly.*

## **Methods**

This section is well presented. However, I have little concern about snake identification. Ashar et al. (2016) established a Remote Envenomation Consultation Service, which authors used during this cohort study. Are these identification based on morphometric analysis or both morphometric as well as DNA barcode analyses? Reliable methods used while identifying the snakes involved in bite to human subject could make this section more convincing. It is because several pitviper species have species complexes. Aren't there species complex in pitviper of Malaysia?

[Ashar AM, Lam MC, Zainudin S, Ismail AK. 2016. A preliminary study on the decision support mobile application for remote snakebite management consultation in Malaysia. Pages 020086-020081–020086. AIP Conference Proceedings: American Institute of Physics Publishing.]

***Response:*** *Thank you for the comment. However, the study was inappropriately quoted. Ashar et.* *al reported on the possibility of developing an online application for existing RECS. RECS was establised in 2012. Ref: http://mstoxinology.blogspot.com/p/recs.html*

## **Results**

**Table 1. Demographic**

please, use frequency instead of symbol f or n, percent instead of % .... Or, make subheadings of this table like in Table 2.

***Response:*** *Thank you. Changes have been made.*

## **Discussion**

Line 159–162: "... bite cases in this study involved local citizens and occurred during non occupational activity possibly because pit vipers are mostly nocturnal and outdoor are mainly performed during daytime. It is uncommon to find pit vipers entering houses, and in this study only 2.9 % of cases occurred indoors."

Rev: [Reid and Theakston (1983)](bookmark://_ENREF_1) mentioned that a prospective hospital-based study of snakebite cases in Malaysia confirmed that snake bite is occupational hazard mainly among males during daylight particularly in a rural Malaysia. But, your findings are contrary to this report which you did not discuss well in the discussion section in support of previous reports and recent findings which are in favour of your report.

***Response:*** *The study quoted above was not properly referred in the report Reid and Theakston (1983). Most likely the study mentioned by them was undifferentiated to the snake family. Thus, concluding the above. However, this study is focused on the pit viper bites in Malaysia. Most of the pit vipers in Malaysia are arboreal and belonging to the Trimeresurus complex species. This study showed that the upper limbs were more frequently involved than the lower limbs.* *This was probably because most of the patients were actively using their hands during farming and gardening close to the ground or above ground. This is in contrast to earlier studies on snake-bite in Malaysia that identified the lower limbs to be more frequently involved [2-8]. This contradiction may be influenced by different snake species involved. The involvement of upper limbs in this study may have been influenced by the activities prior to the incident and the locomotion of the snakes. The common species identified in the other studies were Naja kaouthia, Naja sumatrana, and Calloselasma rhodostoma. These species inhabit lowland forest with an elevation of less than 820m above sea level and frequently found in open environment such as agricultural fields and plantations. They are mainly terrestrial in nature and the cobras are fast moving snakes that can rear up one-third of its body length. The majority of bites from these species were found to be accidental in nature either by stepping on the snake, getting too close to it or did not move away fast enough.*

Line 142-143: "... the actual burden because many victims never reach primary care facilities and are therefore unreported." Rev: If so, where do they receive treatment services? Do they visit to traditional healers like in other Asian and African countries? Please, mention it following "....unreported.".

***Response:*** *Thank you for the query. We do not have any reference of previous study in Malaysia regarding where patients received treatment services. However, unpublished research on prehospital care and health seeking behaviour following a snake bite in Malaysia from 2019-2020 showed only 0.4% visited traditional healer before going to hospital and 10.1% visited traditional healer after being discharged from hospital.*

Line 144: "... The Malaysian literature on snakebite epidemiology is scarce [14].".

Rev: please replace "... The Malaysian literature on snakebite epidemiology is scarce [14]."

With "... The Malaysian literature on proven snakebite epidemiology is scarce [14]."

Line 200: Please, insert full stop and They between "*Bitis arietans*" and "have been referred to" .

***Response:*** *Thank you, changes have been made.*

## **Referencing**

Line 282

"18. Ismail AK, Adnan A, Hassan A, Mohamed Z, Daud R. RECS-01-01. 2014.". Please, make this reference complete with title having full form of the abbreviation RECS- is this original title or did you abbreviate it?

Reid, H. A., & Theakston, R. D. G. (1983). The management of snake bite. *Bulletin of the World Health Organization, 61*(6), 885–895.

***Response:*** *Thank you, the reference for RECS has been changed to a website reference. Malaysian Society on Toxinology [Internet] 2014 [updated 2023: cited 2023 June] Available from: http://mstoxinology.blogspot.com/p/recs.html*

**Reviewer 3**

Conclusions are made in line with the findings. The proposed use of one of the antivenom for GPAV (which is a mono-specific antivenom) for the diverse pit viper species should be supported by previous works that show cross-reactivity and cross-neutralization at least for some of the species implicated in the cases reported.
***Response:*** *Thank you, the necessary references were provided.*

Authors reported a study entitled: Frequency, Geographical Distribution and Outcomes of Pit Viper Bite Envenomation in Malaysia. This is an important work as data on snakebite envenomation is lacking in the country. However, there are several issues with the write-up which authors should try to address for improvement.
***Response:*** *Thank you.*

Comment: From the public health perspective, this is not a typical epidemiological study and is solely based on consultancy from doctors/hospitals to a group of network (RECS), there is a confusion over how representative of the overall data is for the whole country (Malaysia), as suggested by the title. It is good that authors acknowledged this a limitation (see section on limitation). However, the following suggestions should be addressed:

(1) For clarity, the title should be modified accordingly to reflect the nature of the work. May include "analysis based on 523 pit viper envenomation cases consulted to RECS (2017-2020)" as part of it.

***Response:*** *Thank you, the title has been modified to include ‘consulted to Remote Envenomation Consultation Service (RECS) from 2017-2020’*

(2) The "outcomes" is not well defined. Was this referred to the clinical outcome as in both syndrome/toxicity and treatment? Readers from afar would be expecting to read these, naturally as the outcomes of those cases following consultancy provided by the RECS team. The article in fact did not report in depth the clinical manifestations of the bites by the respective species, and no much information was provided on the treatment aspect (for instance, how many vials of GPAV in average, for each species). These should be considered as part of important data to substantiate this article. Introduction is adequate. Methodology is straight forward.

***Response:*** *Thank you, the outcome of this study is from the use of antivenom, surgical intervention and mortality. The number of each antivenom used for each year of study is represented in figure 4, no average of antivenom vials was calculated. However, it was stated in the results that the most patients required only one dose (3 vials) of antivenom.*

Line 89-90: Data was collected following the approval of institutional research ethics committee and RECS. -- Please provide name of institution/IRB and ethics code.
***Response:*** *Thank you, the details has been added in the methodology.*

**Results, discussion and conclusion**

Line 68-72 and geographical map of pit viper cases consulted to RECS show there are very diverse species of pit vipers responsible for SRI and envenomation in Malaysia, and proposed the use of mainly two antivenoms imported from Thailand for use against these indigenous species in Malaysia. The use of CRAV is clearly for the Malayan pit viper as authors rightly explained. However, GPAV is proposed by authors as the antivenom to be used for other species? See also: Line 129-131, Line 167-170. Although many of these species are given the genus Trimeresurus, Malhotra et al. has shown the phylogenetic divergence in them, for example, there are genus or subgenus like Popeia and Craspedocephalus and Parias etc. I think there are also a number of studies showed the variation in their venom content and toxicity. Authors should include the outcome findings of the treatment (they did mention for T. purpureomaculatus, treated with GPAV 3 vials) for the various species consulted to them. It is understood that clinical trial is almost impossible but there have been several experimental or preclinical studies in animals that demonstrated the cross-reactivity or cross-neutralisation activity of GPAV for the various Trimeresurus pit vipers, in particular T. purpureomaculatus since this is the most common biting pit viper species in their consultancy record. These studies should be referenced in making the call for the clinical use of GPAV for the non-species specific bites.
***Response:*** *Thank you, the Trimeresurus complex species has been well documented in Malaysia and it is true that based on clinical testing for cross neutralization has been published and are quoted in the Ministry of Health guidelines and several other references provided.*
