# Supplementary material for: Frequency, geographical distribution and outcomes of pit viper bites in Malaysia consulted to Remote Envenomation Consultancy Services (RECS) from 2017 to 2020
Source: PLoS Negl Trop Dis. 2023 Aug 16;17(8):e0011569. doi: 10.1371/journal.pntd.0011569 (PMC10461839; doi:10.1371/journal.pntd.0011569)
Supplement: Attachment — Submitted filename: PNTD-D-23-006014_R1_230711.docx [file pntd.0011569.s005.docx]

# Review Comments to the Editor

I reviewed this manuscript again thoroughly. This manuscript can be accepted with a minor revision in Discussion and Conclusion sections. Since this article addresses the knowledge gap on expert identified pitviper bites in Malaysia and variations in distribution patterns suggesting antivenom supply, use and stock accordingly, this article can be highly useful to manage snakebite in Malaysia. Further, this paper can also be a reference for antivenom design and distribution because all pit viper bites in this country does not need antivenom therapy. So, this paper is under the scope of PLOS Negl Trop Dis. This paper looks to be outstanding in its discipline for Malaysia where policy makers can use information on proven pitviper bite envenomation and outcomes to improve management of snakebites. They have slightly improved methodology which is sufficient to reproduce (if needed). Overall, this manuscript is well organized and well written. However, authors need to work on this manuscript slightly. I have mentioned my feedbacks to authors in a separate section so that authors will be able to improve this manuscript.

# Review Comments to the Author

# General

Authors have improved this manuscript. However, they have to improve discussion and conclusion sections.

Reid and Theakston (1983) mentioned that a prospective hospital-based study of snakebite cases in Malaysia confirmed that snake bite is occupational hazard mainly among males during daylight particularly in a rural Malaysia. This review article would have cited "Reid HA, Thean P, Martin W. 1963. Epidemiology of snake bite in north Malaya. British Medical Journal 1: 992–997." But, they missed to cite it in their review article. I agreed it with the argument of authors of this manuscript about this article citation. To improve the discussion, however, I would like to suggest authors to refer to Reid et al. 1963 (instead of Reid and Theakston 1983) in order to discuss about their contrasting findings of majority of non-occupational hazards. Authors need to discuss well in the discussion section in support of Reid et al. 1963 and other similar literature (if any). It is important because snakebites are widely understood as an occupational health hazard. Are nocturnal behavior of pitvipers and industrialization of Malaysia responsible for this differential finding in this study? Has the agriculture-based Malaysia during 1960s been changed to Industry-based Malaysia during this study period? If yes, discuss on it in reference to changes in agricultural practices ... most bites might have occurred during hiking, ..., touristic visits, night-camping, etc.

Next, Figure 2 clearly shows that this study data set represent 13 states of the Malaysia. This nationwide survey data is the strength of this study. So, I would like to suggest rephrasing the title of this study as **"Frequency, geographical distribution and outcomes of Pit Viper species bites in Malaysia during 2017 to 2020".** The "Consulted to Remote Envenomation Consultancy Services (RECS)" has been described well in the methodology section (I think this section is not needed in the title).

# Specific

# Line 6: ... Zainalabidin Mohamed@Ismail^3^,

# Please, remove @ and place a space.

# Line 33: .... Indigenous species

# Please, insert "pit viper" between indigenous and species.

# Line 45-46: "... and the geographical distribution is not homogenous."

# Please, replace above with "... and their geographical distribution is not homogenous."

Line 77: Please, replace ".... *Garthius chaseni, Ovophis convictus.*"

with "... *Garthius chaseni,* and *Ovophis convictus.*".

Line 776-77: ... Naja kaouthia, Naja sumatrana, and Calloselasma rhodostoma.

Please, italicize all generic and species name herein and elsewhere.

## Introduction

This section still needs minor revisions. I have suggested you minor improvement below specifically.

**Line 52-54:**

1. Patikorn C, Leelavanich D, Ismail AK, Othman I, Taychakhoonavudh S, Chaiyakunapruk N. 2020. Global systematic review of cost of illness and economic evaluation studies associated with snakebite. Journal of Global Health 10: 1–8.

2. Patikorn C, Ismail AK, Abidin SAZ, Blanco FB, Blessmann J, Choumlivong K, Comandante JD, Doan UV, Mohamed Z, Khine YY. 2022. Situation of snakebite, antivenom market and access to antivenoms in ASEAN countries. BMJ Global Health 7: e007639.

3. Patikorn C, Ismail AK, Zainal Abidin SA, Othman I, Chaiyakunapruk N, Taychakhoonavudh S. 2022. Potential economic and clinical implications of improving access to snake antivenom in five ASEAN countries: A cost-effectiveness analysis. PLOS Neglected Tropical Diseases 16: e0010915.

4. Patikorn C, Blessmann J, Nwe MT, Tiglao PJG, Vasaruchapong T, Maharani T, Doan UV, Zainal Abidin SA, Ismail AK, Othman I. 2022. Estimating economic and disease burden of snakebite in ASEAN countries using a decision analytic model. PLoS Neglected Tropical Diseases 16: e0010775.

\## The reference number 1 and 3 discuss mainly about economic burden due to snakebites. Since reference number 2 and 4 have discussed about incidence of snakebites illustrating the snakebite burden in terms of number of affected people due to snakebites, I would like to suggest keeping only reference number 2 and 3.

## Results

In Table 2, please, list the species of snakes according to ascending or descending order of respective frequency or percent in order to the pick up the species most or least involve in human bites quickly.

## Discussion

Please, improve the first paragraph of the discussion by highlighting your major findings:

I would like to suggest you to write it as: "In our knowledge, this is the second study on the incidence and geographical distribution of pit viper species based on confirmed snakebite cases presenting to Malaysian hospitals although snake-related injuries are common in Malaysia (references:

1) Reid HA, Thean P, Martin W. 1963. Epidemiology of snake bite in north Malaya. British Medical Journal 1: 992–997.

2) Lim BL, bin Ibrahim AB. 1970. Bites and stings by venomous animals with special reference to snake bites in West Malaysia. Medical Journal of Malaya 25: 128–141.

3) Muthusamy E. 1988. Snake bite: experience in Bukit Mertajam District Hospital, Pulau Pinang. Singapore Medical Journal 29: 383–386.

4) Jamaiah I, Rohela M, Ng T, Ch'ng K, Teh Y, Nurulhuda A, Suhaili N. 2006. Retrospective prevalence of snakebites from Hospital Kuala Lumpur (HKL)(1999-2003). Southeast Asian Journal of Tropical Medicine and Public Health 37: 200–205.

5) Chew KS, Khor HW, Ahmad R, Rahman NH. 2011. A five-year retrospective review of snakebite patients admitted to a tertiary university hospital in Malaysia. International Journal of Emergency Medicine 4: 41.

6) Chen HL, Chuah YQ, Eng KL, Michelle L, Yeoh Y, Binti AR. 2019. Use of snake antivenom and clinical outcomes in snake envenomation: A retrospective study in a Tertiary Hospital in Penang, Malaysia. Asia Pacific Journal of Medical Toxicology 8.). Recently, Ismail et al. (2023) reported the envenomations and outcomes of proven *Tropidolaemus* species bites in Sabah and Sarawak and Peninsular Malaysia (reference: Ismail AK, Abd Hamid MNH, Ariff NA, Frederic Ng VER, Goh WC, Abdul Samat NS, Osman AMZ, Safferi RS, Mohamed Ismail Z. 2023. Frequency, clinical characteristics and outcomes of *Tropidolaemus* species bite envenomations in Malaysia. PLoS Neglected Tropical Diseases 17: e0010983.). Similar, documentation of envenomation due bites of snakes identified by experts is needed in this country and elsewhere affected by snakebites. The robust statistical data on snakebite envenomation is lacking and cases reported to health ministry by clinics and hospitals are an underestimate of the actual burden because many victims never reach primary care facilities [25,26]. Further, the Malaysian literature on proven snakebite epidemiology is scarce [12,17]. These are challenges to assess true impact snakebite envenomation in Malaysia. In a study by Chew, 260 cases recorded from a teaching hospital, 52.9% were unidentified. The most common venomous snakebites were only documented as caused by cobras 152 (*n*=64, 25%) and by pit vipers (*n*=52, 20%) [5]. Our report of incidence and geographical distribution of expert-identified pit viper species can be implied for the proper snakebite management in Malaysia."

I would like to suggest removing the second paragraph because this study is solely concentrated to pit viper bites, not colubrid snakebites. The second paragraph discusses about the venomous colubrids which looks irrelevant to the findings in this study. Rather, authors can develop this paragraph as: "Our checklist of pitviper species (Table 2) corresponds to the recent review of the terrestrial venomous snakes of Malaysia including those from the states of Sabah and Sarawak [15]. Overall, in addition to the viperid snakes, elapids and some colubrid snake species (for example, the Red-necked keelback, *Rhabdophis subminiatus* which can cause severe coagulopathy [12,17]) cause life-threatening envenomation to Malaysians."

Then, you address following comments on the discussion:

# A) Table 2 and Figure 3 are missing *T. malcomi*, Trimeresurus buniana. In introduction, these snakes were highlighted to be distributed commonly in Malaysia. Please, discuss about missing of these snake species in the dataset of identified snakes involved in human bites in this country.

**B) Next, t**he reference number 10 (Reid et al. 1963) mentions that *Calloselasma rhodostoma* (previously, it was aka *Ancistrodon rhodostoma*) was the most common medically relevant snakes in Malaysia. Similar prevalence was reported by E Muthusamy (1988) in SNAKE BITE: EXPERIENCE IN BUKIT MERTAJAM DISTRICT HOSPITAL, PULAU PINANG SING MED J. 1988; 29: 383-386. But, this study in Malaysia found that the *Trimeresurus purpureomaculatus* is the most common medically relevant snakes in this country. Why? Please, elaborate it in the discussion section.

Line 156 - should be "and Viperidae. However,"

Line 158 - please, add comma after the example as "...for example, the..."

## Conclusion

I would like to suggest authors to reduce the length of conclusion including suggestions to policy makers and further studies.

My suggestions:

Pit viper bite is common but neglected SRI in Malaysia where a distinct variation in distribution patterns of pit viper species exists. This distinction and mild envenomation due to *Tropidolaemus* species bite requiring no antivenom therapy suggests distributing, stock, and using antivenom in hospitals in different states of Malaysia judiciously. There is importance of similar and appropriately designed study to record and document proven snakebites due to other snake family distributed in Malaysia. This study finding also promotes appropriate health seeking awareness and optimal patients’ care consulting with the relevant experts.

## Referencing

Line 262

Please, remove @ from "reference number 2. in reference section. What does @ mean herein? I think it is erroneous insertion. Does it have any meaning in the name of author?

Please, be consistent while naming the journals throughout the reference section. For example, the reference number 4 and 5 have different naming styles (4: PLOS Neglected Tropical Diseases, and 5: Int J Emerg Med.).

Please, remove "null" from the reference number 7 "Lim BL, bin Ibrahim AB. 1970. Bites and stings by venomous animals with special reference to snake bites in West Malaysia. Medical Journal of Malaya 25: 128–141.".

Please, italicize all Generic and Specific names elsewhere. For example, in the reference number 19, please, italicize "Trimeresurus".

Reid, H. A., & Theakston, R. D. G. (1983). The management of snake bite. *Bulletin of the World Health Organization, 61*(6), 885–895.
