# Supplementary material for: Frequency, geographical distribution and outcomes of pit viper bites in Malaysia consulted to Remote Envenomation Consultancy Services (RECS) from 2017 to 2020
Source: PLoS Negl Trop Dis. 2023 Aug 16;17(8):e0011569. doi: 10.1371/journal.pntd.0011569 (PMC10461839; doi:10.1371/journal.pntd.0011569)
Supplement: Attachment — Submitted filename: Response R1.docx [file pntd.0011569.s006.docx]

**RESPONSES TO REVIEWER**

Thank you for the comments and suggestions. Below are the responses to the reviewers.

**Reviewer 1**

Most of the previous queries have been addressed. But, there are minor corrections as follows,

Line 52 ASEAN ?

*Response: Thank you for the comment. We have changed ASEAN to “South-East Asian countries”.*

Line 64 snake bite snakebite (one word)

*Response: Thank you for the comment. The changes have been made accordingly.*

Line 71-74

It is better to include common name of these species in brackets or common name then scientific name in brackets. Also, mention the endemic species a recent paper describes on taxonomic updates of Green pit vipers. Please see whether these names (genus) are changed or not.

*Response: Thank you for the comment. We prefer to maintain as the scientific names because common names may differ between cultures. All the current scientific names are updated accordingly based on the references list (no 13, 14 and 16).*

Results: Only % has been mentioned. But, number should be there with % as n (%).

*Response: Thank you for the comment. We have inserted the n for each percentage mentioned in the results.*

Line 102 56.7%3?

*Response: Thank you for the comment. We have corrected accordingly.*

Line 104 Median value should follow IQR

*Response: Thank you for the comment. The median IQR value has been included.*

Line 76-77 scientific names should be italic

*Response: Thank you for the comment. It was already in italic.*

**Reviewer 2**
Authors have improved this manuscript. However, they have to improve discussion and conclusion sections.
Reid and Theakston (1983) mentioned that a prospective hospital-based study of snakebite cases in Malaysia confirmed that snake bite is occupational hazard mainly among males during daylight particularly in a rural Malaysia. This review article would have cited "Reid HA, Thean P, Martin W. 1963. Epidemiology of snake bite in north Malaya. British Medical Journal 1: 992–997." But, they missed to cite it in their review article. I agreed it with the argument of authors of this manuscript about this article citation. To improve the discussion, however, I would like to suggest authors to refer to Reid et al. 1963 (instead of Reid and Theakston 1983) in order to discuss about their contrasting findings of majority of non-occupational hazards. Authors need to discuss well in the discussion section in support of Reid et al. 1963 and other similar literature (if any). It is important because snakebites are widely understood as an occupational health hazard. Are nocturnal behavior of pitvipers and industrialization of Malaysia responsible for this differential finding in this study? Has the agriculture-based Malaysia during 1960s been changed to Industry-based Malaysia during this study period? If yes, discuss on it in reference to changes in agricultural practices ... most bites might have occurred during hiking, ..., touristic visits, night-camping, etc.

*Response: Thank you for the noticing the original reference by Reid et al 1963. This article is already cited in our reference no 10 and the deficiencies of this study along with the other earlier studies have been clearly mentioned from line 54 – 60 in the introduction justifying the importance of the current studies. Yes, a lot has changed from 1960s Malaysia to the current period of this study. This study does not include all the other snake species, however the scope of this study did identify the geographical distribution of various pit viper species in Malaysia in accordance to the current IUCN distribution of pit viper species in Malaysia. The current study does not identify hiking activities or tourists visits or camping etc to be a major contributor to the number of bites from pit viper in Malaysia. As mentioned in the previous response, another unpublished study on Cameron Highlands, a major tourist and nature activities do not record significant number of bites among these groups of visitors. Further comments regarding the possible differences in the involvement of bitten area has been well discussed in the discussion line 163 – 183.*

Next, Figure 2 clearly shows that this study data set represent 13 states of the Malaysia. This nationwide survey data is the strength of this study. So, I would like to suggest rephrasing the title of this study as "Frequency, geographical distribution and outcomes of Pit Viper species bites in Malaysia during 2017 to 2020". The "Consulted to Remote Envenomation Consultancy Services (RECS)" has been described well in the methodology section (I think this section is not needed in the title).

*Response: Thank you for the suggestion, however as the reviewer has demonstrated there are still confusion regarding the scope of study, therefore we maintained the title which include both Malaysia and consultation to RECS to be extra clear from the start.*

Specific

Line 6: ... Zainalabidin Mohamed@Ismail3,

Please, remove @ and place a space.

*Response: Thank you. This is not an anomaly. This is the formal name of the author.*

Line 33: .... Indigenous species

Please, insert "pit viper" between indigenous and species.

*Response: Thank you.*

Line 45-46: "... and the geographical distribution is not homogenous."

Please, replace above with "... and their geographical distribution is not	homogenous."

*Response: Thank you. Changes have been made.*

Line 77: Please, replace ".... Garthius chaseni, Ovophis convictus."

with "... Garthius chaseni, and Ovophis convictus.".

*Response: Thank you. Changes have been made.*

Line 176-177: ... Naja kaouthia, Naja sumatrana, and Calloselasma rhodostoma.

Please, italicize all generic and species name herein and elsewhere.

*Response: Thank you. Changes have been made.*

Introduction

This section still needs minor revisions. I have suggested you minor improvement below specifically.

Line 52-54:
1. Patikorn C, Leelavanich D, Ismail AK, Othman I, Taychakhoonavudh S, Chaiyakunapruk N. 2020. Global systematic review of cost of illness and economic evaluation studies associated with snakebite. Journal of Global Health 10: 1–8.

2. Patikorn C, Ismail AK, Abidin SAZ, Blanco FB, Blessmann J, Choumlivong K, Comandante JD, Doan UV, Mohamed Z, Khine YY. 2022. Situation of snakebite, antivenom market and access to antivenoms in ASEAN countries. BMJ Global Health 7: e007639.

3. Patikorn C, Ismail AK, Zainal Abidin SA, Othman I, Chaiyakunapruk N, Taychakhoonavudh S. 2022. Potential economic and clinical implications of improving access to snake antivenom in five ASEAN countries: A cost-effectiveness analysis. PLOS Neglected Tropical Diseases 16: e0010915.

4. Patikorn C, Blessmann J, Nwe MT, Tiglao PJG, Vasaruchapong T, Maharani T, Doan UV, Zainal Abidin SA, Ismail AK, Othman I. 2022. Estimating economic and disease burden of snakebite in ASEAN countries using a decision analytic model. PLoS Neglected Tropical Diseases 16: e0010775.

\## The reference number 1 and 3 discuss mainly about economic burden due to snakebites. Since reference number 2 and 4 have discussed about incidence of snakebites illustrating the snakebite burden in terms of number of affected people due to snakebites, I would like to suggest keeping only reference number 2 and 3.

*Response: Thank you for the suggestions, however we believed that all four references are related and formed a better representation to the sentence it refers to which aso includes the incidence, mobidity and mortality and access to antivenom.*

Results

In Table 2, please, list the species of snakes according to ascending or descending order of respective frequency or percent in order to the pick up the species most or least involve in human bites quickly.

*Response: Thank you for the suggestion. This table is designed according to the genus and species for better differentiation. The significance of the results in terms of numbers and percentages has been highlighted in the results paragraph.*

Discussion

Please, improve the first paragraph of the discussion by highlighting your major findings:

I would like to suggest you to write it as: "In our knowledge, this is the second study on the incidence and geographical distribution of pit viper species based on confirmed snakebite cases presenting to Malaysian hospitals although snake-related injuries are common in Malaysia (references:

1) Reid HA, Thean P, Martin W. 1963. Epidemiology of snake bite in north Malaya. British Medical Journal 1: 992–997.

2) Lim BL, bin Ibrahim AB. 1970. Bites and stings by venomous animals with special reference to snake bites in West Malaysia. Medical Journal of Malaya 25: 128–141.

3) Muthusamy E. 1988. Snake bite: experience in Bukit Mertajam District Hospital, Pulau Pinang. Singapore Medical Journal 29: 383–386.

4) Jamaiah I, Rohela M, Ng T, Ch'ng K, Teh Y, Nurulhuda A, Suhaili N. 2006. Retrospective prevalence of snakebites from Hospital Kuala Lumpur(HKL)(1999-2003). Southeast Asian Journal of Tropical Medicine and Public Health 37: 200–205.

5) Chew KS, Khor HW, Ahmad R, Rahman NH. 2011. A five-year retrospective review of snakebite patients admitted to a tertiary university hospital in Malaysia. International Journal of Emergency Medicine 4: 41.

6) Chen HL, Chuah YQ, Eng KL, Michelle L, Yeoh Y, Binti AR. 2019. Use of snake antivenom and clinical outcomes in snake envenomation: A retrospective study in a Tertiary Hospital in Penang, Malaysia. Asia Pacific Journal of Medical Toxicology

*Response: Thank you for the suggestion. Changes has been made. All the references above have already been documented in the reference list. Note that there was no previous documented study specifically reviewing the pit viper bite envenomation in Malaya or Malaysia.*

8.). Recently, Ismail et al. (2023) reported the envenomations and outcomes of proven Tropidolaemus species bites in Sabah and Sarawak and Peninsular Malaysia (reference: Ismail AK, Abd Hamid MNH, Ariff NA, Frederic Ng VER, Goh WC, Abdul Samat NS, Osman AMZ, Safferi RS, Mohamed Ismail Z. 2023. Frequency, clinical characteristics and outcomes of Tropidolaemus species bite envenomations in Malaysia. PLoS Neglected Tropical Diseases 17: e0010983.). Similar, documentation of envenomation due bites of snakes identified by experts is needed in this country and elsewhere affected by snakebites. The robust statistical data on snakebite envenomation is lacking and cases reported to health ministry by clinics and hospitals are an underestimate of the actual burden because many victims never reach primary care facilities [25,26]. Further, the Malaysian literature on proven snakebite epidemiology is scarce [12,17]. These are challenges to assess true impact snakebite envenomation in Malaysia. In a study by Chew, 260 cases recorded from a teaching hospital, 52.9% were unidentified. The most common venomous snakebites were only documented as caused by cobras 152 (n=64, 25%) and by pit vipers (n=52, 20%) [5]. Our report of incidence and geographical distribution of expert-identified pit viper species can be implied for the proper snakebite management in Malaysia."

*Response: Thank you. Changes has been made in the first paragraph of the discussion.*

I would like to suggest removing the second paragraph because this study is solely concentrated to pit viper bites, not colubrid snakebites. The second paragraph discusses about the venomous colubrids which looks irrelevant to the findings in this study. Rather, authors can develop this paragraph as: "Our checklist of pitviper species (Table 2) corresponds to the recent review of the terrestrial venomous snakes of Malaysia including those from the states of Sabah and Sarawak [15]. Overall, in addition to the viperid snakes, elapids and some colubrid snake species (for example, the Red-necked keelback, Rhabdophis subminiatus which can cause severe coagulopathy [12,17]) cause life-threatening envenomation to Malaysians."

*Response: Thank you. Paragraph 2 of the discussion has been revised.*

Then, you address following comments on the discussion:

A) Table 2 and Figure 3 are missing T. malcomi, Trimeresurus buniana. In introduction, these snakes were highlighted to be distributed commonly in Malaysia. Please, discuss about missing of these snake species in the dataset of identified snakes involved in human bites in this country.

*Response: Thank you for the suggestion, we have added a short comment in the result and discussion as there were no cases from both species during the study period, but that does not mean that these two species are not present in Malaysia and the reason being T. buniana is found only on Tioman island and T. malcomi are found on the highlands of Sabah and Sarawak.*

B) Next, the reference number 10 (Reid et al. 1963) mentions that Calloselasma rhodostoma (previously, it was aka Ancistrodon rhodostoma) was the most common medically relevant snakes in Malaysia. Similar prevalence was reported by E Muthusamy (1988) in SNAKE BITE: EXPERIENCE IN BUKIT MERTAJAM DISTRICT HOSPITAL, PULAU PINANG SING MED J. 1988; 29: 383-386. But, this study in Malaysia found that the Trimeresurus purpureomaculatus is the most common medically relevant snakes in this country. Why? Please, elaborate it in the discussion section.

*Response: Thank you. The reason behind the discrepancy has been mentioned. Not all pit vipers are present homogenously throughout the country and for these two reports the study population is limited to hospitals in the Northern states of Peninsular Malaysia which predominantly occupied by the Malayan pit viper. Secondly, this study involves consultations to RECS. Not all snakebite patients who presented to the hospitals in the country are included in this study.*

Line 156 - should be "and Viperidae. However,"

*Response: Thank you. Changes has been made.*

Line 158 - please, add comma after the example as "...for example, the..."

*Response: Thank you. Changes has been made.*

Conclusion

I would like to suggest authors to reduce the length of conclusion including suggestions to policy makers and further studies.

My suggestions:

Pit viper bite is common but neglected SRI in Malaysia where a distinct variation in distribution patterns of pit viper species exists. This distinction and mild envenomation due to Tropidolaemus species bite requiring no antivenom therapy suggests distributing, stock, and using antivenom in hospitals in different states of Malaysia judiciously. There is importance of similar and appropriately designed study to record and document proven snakebites due to other snake family distributed in Malaysia. This study finding also promotes appropriate health seeking awareness and optimal patients’ care consulting with the relevant experts.
*Response: Thank you. Changes has been made.*

Referencing

Line 262

Please, remove @ from "reference number 2. in reference section. What does @ mean herein? I think it is erroneous insertion. Does it have any meaning in the name of author?

*Response: Thank you. It is the formal name of the author.*

Please, be consistent while naming the journals throughout the reference section. For example, the reference number 4 and 5 have different naming styles (4: PLOS Neglected Tropical Diseases, and 5: Int J Emerg Med.).

*Response: Thank you. Changes has been made.*

Please, remove "null" from the reference number 7 "Lim BL, bin Ibrahim AB. 1970. Bites and stings by venomous animals with special reference to snake bites in West Malaysia. Medical Journal of Malaya 25: 128–141.".

*Response: Thank you. Changes has been made.*

Please, italicize all Generic and Specific names elsewhere. For example, in the reference number 19, please, italicize "Trimeresurus".

*Response: Thank you. Changes has been made.*

Reid, H. A., & Theakston, R. D. G. (1983). The management of snake bite. Bulletin of the World Health Organization, 61(6), 885–895.

*Response: Thank you. The reference that was included was no 10: Reid HA, Thean PC, Martin WJ. Epidemiology of Snake Bite in North Malaya. BMJ. 1963;1: 992–997.*

**Reviewer 3**

In paragraphs line 196-202, and according to authors response to one reviewer's comment that says "The proposed use of one of the antivenom for GPAV (which is a mono-specific antivenom) for the diverse pit viper species should be supported by previous works that show cross-reactivity and cross-neutralization at least for some of the species implicated in the cases reported." Authors responded that the necessary references were provided without specifying them. To another comment by the reviewer, authors responded "cross neutralization has been published and are quoted in the Ministry of Health guidelines and several other references provided." However, we could not identify these references in the revised version. The previous studies are obvious in the literature, a large body of studies on cross-neutralization of GPAV (Trimeresurus albolabris mono antivenom) with efficacy testing for various Trimeresurus pitviper species, including wiroti, nebularis, purpureomaculatus, sumatranus etc (these species were documented in table 2) have been reported for Malaysian venoms. Perhaps the authors have overlooked the references in their revision, but they would be crucial to support the outcome reported and the proposed use of monovalent GPAV for other pitviper species (except malayan pitvper) in Malaysia.

*Responses: Thank you for the comment. We have included the reference (no 17, 28) that is used for the green pit viper antivenom in Malaysia. The use of GPAV for the trimeresurus complex species in Malaysia was supported by the evidence mentioned in the Ministry of Health guidelines. This study reflects the outcome of the clinical practice based on what is guided by the Malaysian Ministry of Health guidelines. We acknowledged there are more recent studies regarding cross neutralization of the GPAV against various green pit viper venom in Malaysia. This will be included in the upcoming updates of the Malaysian Ministry of Health guidelines.*
